# Insights into non-informative results from non-invasive prenatal screening through gestational age, maternal BMI, and age analyses

**DOI:** 10.1371/journal.pone.0280858

**Published:** 2024-03-07

**Authors:** Juraj Gazdarica, Natalia Forgacova, Tomas Sladecek, Marcel Kucharik, Jaroslav Budis, Michaela Hyblova, Martina Sekelska, Andrej Gnip, Gabriel Minarik, Tomas Szemes

**Affiliations:** 1 Faculty of Natural Sciences, Comenius University, Bratislava, Slovakia; 2 Geneton Ltd., Bratislava, Slovakia; 3 Slovak Centre of Scientific and Technical Information, Bratislava, Slovakia; 4 Comenius University Science Park, Bratislava, Slovakia; 5 Institute of Clinical and Translational Research, Biomedical Research Center, Slovak Academy of Sciences, Bratislava, Slovakia; 6 Trisomy test Ltd., Nitra, Slovakia; 7 Medirex Inc., Bratislava, Slovakia; Xuzhou Maternity and Child Health Care Hospital affiliated to Xuzhou Medical UniversityCare Hospital Affiliated to Xuzhou Medical University, CHINA

## Abstract

The discovery of cell-free fetal DNA fragments in the maternal plasma initiated a novel testing method in prenatal care, called non-invasive prenatal screening (NIPS). One of the limitations of NIPS is the necessity for a sufficient proportion of fetal fragments in the analyzed circulating DNA mixture (fetal fraction), otherwise, the sample is uninterpretable. We present the effect of gestational age, maternal body mass index (BMI), and maternal age on the fetal fraction (FF) of the sample. We retrospectively analyzed data from 5543 pregnant women with a single male fetus who underwent NIPS from which 189 samples received a repeat testing due to an insufficient FF. We showed the relationship between the failure rate of the samples after the repeated analysis, the FF, and the gestational age at the first sampling. Next, we found that different maternal BMI categories affect the FF and thus the chance of an informative redraw. A better understanding of the factors affecting the FF will reduce the number of non-informative calls from repeated analyzes. In this study, we provide helpful information to clinicians on how to approach non-informative analyses.

## Introduction

In the last decade, non-invasive prenatal testing (NIPT), also referred to as non-invasive prenatal screening (NIPS), has been implemented as a leading-edge technique for screening fetal aneuploidy. This approach uses the potential of massively parallel sequencing of cell-free DNA (cfDNA) present in the maternal bloodstream [1,2]. Compared to traditional aneuploidy screening methods (serum- and ultrasound-based test), cfDNA sequencing can detect chromosomal aneuploidies with a high sensitivity of 97.45% to 100% and a specificity of 99.94% to 99.96% [3]. Compared to standard invasive techniques such as Chorionic Villus Sampling (CVS) and amniocentesis, cfDNA testing is non-invasive, easy to perform and poses no direct threat to either the mother or the fetus [4]. For these reasons NIPT has rapidly gained popularity in many countries worldwide as a routine part of health care for pregnant women [5,6].

An inevitable issue with NIPT as a universal screening test is the potential attainment of uninformative (non-reportable, or no-call) results, which may be caused by many factors. The most common cause for test failure is insufficient fetal fraction (FF) that reportedly occurs in approximately 0.1% to 6.1% of NIPT cases depending on the particular clinical center and NIPT technology used [3,5]. Furthermore, some laboratories set a limit on the sequencing depth due to deeper sequencing can usually reduce the failure rate considerably.

There are basically several ways in which FF estimates can be obtained such as single nucleotide polymorphism (SNP)-based methods, whole genome sequencing (WGS)-based methods using various differences between maternal and fetal cell-free DNA, or methods based on sex chromosomes [6]. To provide optimal management of women who undergo NIPT with an initial uninformative result, it is crucial to evaluate the factors associated with the success rate of repeated samplings and analyses. The most well-established and clinically significant factor associated with no-result rates is the maternal weight and/or body mass index (BMI). A review of the literature shows a strong negative correlation between FF and higher maternal BMI [7–16]. The inverse relationship between maternal weight (or BMI) and FF can be explained by a combination of two simultaneous effects, the dilution of a fixed amount of fetal cfDNA into a larger maternal blood volume and an increase in the concentration of maternal circulating cell-free DNA (ccfDNA) with increasing maternal weight [16–19]. A possible explanation for the higher release of maternal cfDNA derived from the active remodeling of adipose tissue leads to increased adipocyte necrosis and stromal vascular apoptosis [20]. Previous studies suggested that the other significant factor, positively correlated with FF levels, is the fetus’s gestational age. It is well known that FF increases with later gestational age [7,21]. There have also been reports of a temporary decrease in FF from the first to the second trimester due to increased maternal weight during this gestational time [22]. It has been postulated that FF is also associated with maternal characteristics such as maternal age, ethnicity, and smoking; for example, a lower FF was observed in older women [13–15] and women of Afro-Caribbean [11], South and East Asian origin [9,13,23], and a higher FF in smokers than non-smokers [11].

Women with uninformative results are often advised to undergo blood redraw and re‐testing, assuming that an increase in the fetus size would lead to a higher FF value and thus a reportable result. However, this brings discomfort and more uncertainty to women and extra costs for laboratories. Furthermore, it was reported that nearly 40–50% of women with a failed NIPT result would receive uninformative results in the repeating testing again, depending on the NIPT methodology used [6]. Therefore, the better understanding of factors influencing NIPT results is crucial for optimal prenatal testing management.

In our previous study, we demonstrated that the FF of female fetuses was significantly higher than male fetuses at the same gestational age, due to the smaller gonosome Y/chromosome Y and biological differences between the sexes. We also observed that the FF does not grow consistently with fetal maturity due to a drop in 15 weeks of gestation [24]. The objective of the present study was to analyze the data of 5543 pregnant women who underwent NIPT in the Slovak population including 189 repeated sampling and following analysis due to insufficient FF of the first sampling. First, we examined the effect of maternal BMI, maternal age and gestational age on the FF. We not only identified which factors negatively influence the success rate of NIPT but, more importantly, whether these factors might affect the chance of an informative redraw. In addition, we demonstrated a model for predicting the success of the second sampling based on known factors from the first sampling.

## Materials and methods

### Sample acquisition

We studied 5543 pregnancies undergoing NIPT with a single male fetus (S1 Dataset), of which 5354 samples were negative after the first sampling (healthy), and 189 samples were uninformative after the first sampling (due to the low FF (<5%)) with consecutive repeated testing (S1 Fig). The time interval of sample collection was from May 2017 to March 2021. The FF, according to the method based on the proportion of fragments on the Y chromosome [7], has been calculated for these samples. Our work was part of two clinical studies approved by the Ethical Committee of the Bratislava Self-Governing Region (Sabinovska ul.16, 820 05 Bratislava): the first one called “NIPT study” (study ID 35900_2015 approved on 30 April 2015 under the decision ID 03899_2015) and the second one called “SNiPT” (study ID 37136/2018 approved on 11 June 2018 under the decision ID 07507/2018/HF). All patients in the study signed written informed consent consistent with the Helsinki declaration, which the ethics mentioned above committee approved. NIPT was performed for common trisomies.

### Sample preparation and sequencing

Blood from pregnant women was collected into EDTA tubes and kept at 4°C temperature until plasma separation. Blood plasma was separated within 36 h after collection and stored at −20 °C unit DNA isolation. DNA was isolated using the Qiagen DNA Blood Mini kit (Qiagen, Germany). The extracted DNA was quantified using a Qubit dsDNA HS Assay Kit (Thermo Fisher Scientific, Oregon, USA). DNA libraries were prepared using the TruSeq Nano DNA Library Prep Kit (Illumina, San Diego, USA) according to the previously published protocol. A minimum limit of library concentration of 0.1 ng/μL was used in subsequent sequencing. A 2100 Bionalyzer (Agilent, Waldbronn, Germany) was used for quality control of final libraries. A NextSeq 500/550 platform and High Output Sequencing Kit v2 (75 cycles) (Illumina) with pair-end sequencing protocol (2 × 35 cycles) were used for sequencing [24–26].

### Statistical analysis

The significance of our findings was evaluated using statistical tests implemented in the Python script package. The linear dependency of the two scores in negative samples was calculated with Pearson correlation.

We performed a logistic regression analysis of factors from maternal and pregnancy characteristics (maternal age, BMI, weight, height, and gestational age) in the prediction of failed testing similarly to a previous study [15]. Multivariate analysis was conducted only on features that were found to be statistically important by the univariate analysis (and BMI was excluded due to high correlation with weight and height). The computation was performed by the *Logit* functionality of *statsmodels* Python package.

We trained 6 classifiers (Logistic regression, SVC, LDA, QDA, RandomForest [27], and XGBoost [28] to predict the success (informative/uninformative) of the second sampling. The dataset of sample attributes such as maternal age, gestational age from the first sampling, BMI, height, weight, the difference between samplings (in days), FF, and DNA library concentration determined from the first analysis were used to classify samples after failure in the first analysis to be informative in the second analysis or not. We divided the dataset into 3 parts: training dataset representing 70% of samples (132, 50 uninformative, and 82 informative), validation set for hyperparameter tuning, and model comparison—constituting 15% of dataset samples (28, 14 uninformative, and 14 informative), and test dataset for final evaluation (29, 13 uninformative and 16 informative). The data was scaled (standardized) prior to modeling. We also tried to predict the FF after second sampling using the same dataset with regularized linear models (Ridge regression implemented in scikit-learn package [27]).

## Results

### Factors affecting fetal fraction

In our study, 189 out of 5543 tests were uninformative due to low FF after the first sampling. We performed univariate logistic analysis of factors that affected the initial uninformative analysis. The logistic analysis showed great concordance with the previous study [15] in all of the studied parameters (Table 1). In the (S1 Table), we also present the logistic analysis for samples with gestational age lower than 14th week as in the previous study [15]. The odds ratios from the multivariate analysis show that maternal weight is the most significant factor and one additional kilogram translates to about a 6.5% increase (or 4.9% in [15]) in risk of uninformative analysis (Table 2).

**Table 1:** Factors affecting cfDNA test failure (univariate analysis).

Variable	Odds Ratio [15]	P [15]	Odds Ratio (our dataset)	P (our dataset)
Maternal age in years	1.048 (1.033–1.064)	<0.0001	1.042 (1.010–1.074)	0.009
Maternal weight in kg	1.041 (1.037–1.044)	<0.0001	1.062 (1.054–1.071)	<0.001
Maternal height in cm	1.001 (0.991–1.011)	0.841	1.009 (0.985–1.034)	0.458
Gestational age in weeks	0.872 (0.824–0.923)	<0.0001	0.868 (0.815–0.926)	<0.001
BMI kg/m^2^	—	—	1.194 (1.166–1.223)	<0.001

**Table 2:** Factors affecting cfDNA test failure (multivariate analysis).

Variable	Odds Ratio [15]	P [15]	Odds Ratio (our dataset)	P (our dataset)
Maternal age in years	1.024 (1.009–1.041)	0.002	1.026 (0.994–1.059)	0.108
Maternal weight in kg	1.049 (1.045–1.053)	<0.0001	1.065 (1.056–1.074)	<0.001
Gestational age in weeks	0.847 (0.792–0.906)	<0.0001	0.833 (0.779–0.890)	<0.001

#### Maternal BMI and age

**Fig 1:**
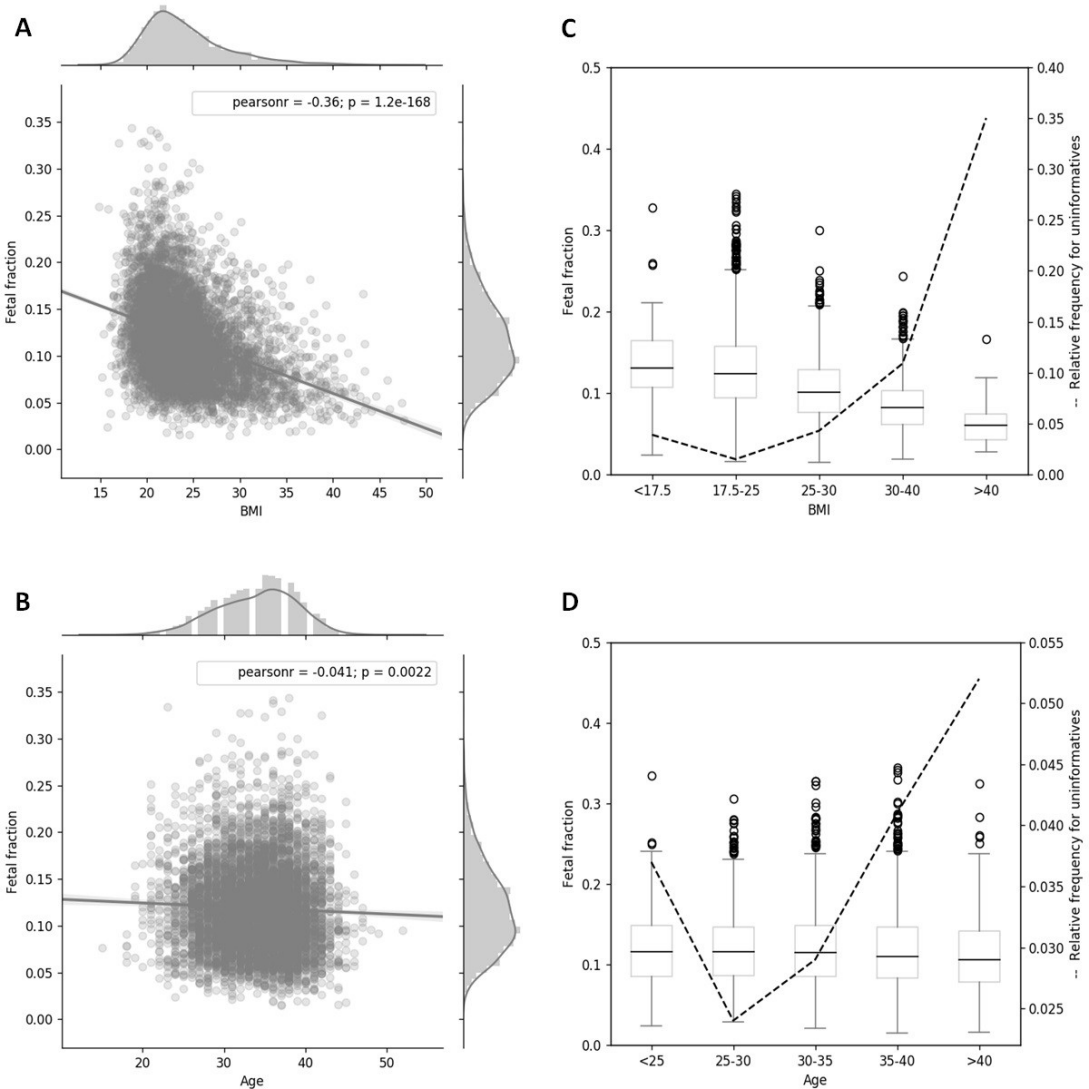
Effect of maternal BMI and maternal age on the fetal fraction. (A) Decrease in fetal fraction with increasing maternal BMI (Pearson’s correlation coefficient = -0.36, p = 1.2e - 168). (B) A slight decrease in fetal fraction with increasing maternal age (Pearson’s correlation coefficient = -0.041, p = 0.0022). (C) The effect of maternal BMI according to the WHO obesity classification groups and relative frequency distribution for uninformative samples. (D) The effect of maternal age on the percentage of fetal fraction across all subjects in the five groups of maternal age and relative frequency distribution for uninformative samples.

Here, we considered all NIPT analyses generated during production with initial FF shown in the (S2 Fig). Using Pearson correlation analysis, it was found that FF in the Slovak population had a negative association with maternal BMI (Pearson’s correlation coefficient = -0.36, p = 1.2e-168) as shown in Fig 1A. The study also evaluated the correlation between FF and maternal age, which revealed an overall trend towards a slightly significant decrease in FF (Pearson’s correlation coefficient = -0.041, p = 0.0022) as presented in Fig 1B. Subsequently, all pregnant women included in our study were categorized into five groups based on the World Health Organization (WHO) obesity classification system standardized for European BMI [29] and five groups of different ages. The median FF and number of informative/uninformative samples for each weight and age class are summarized in (S2 Table). Association between each group (maternal BMI and age) and FF, with relative frequency distribution for uninformative samples, are presented in Fig 1C and 1D. Overall, the results across the BMI and age groups indicate a decline in FF with increasing maternal BMI and age.

### Factors affecting repeated cfDNA test failure

**Fig 2:**
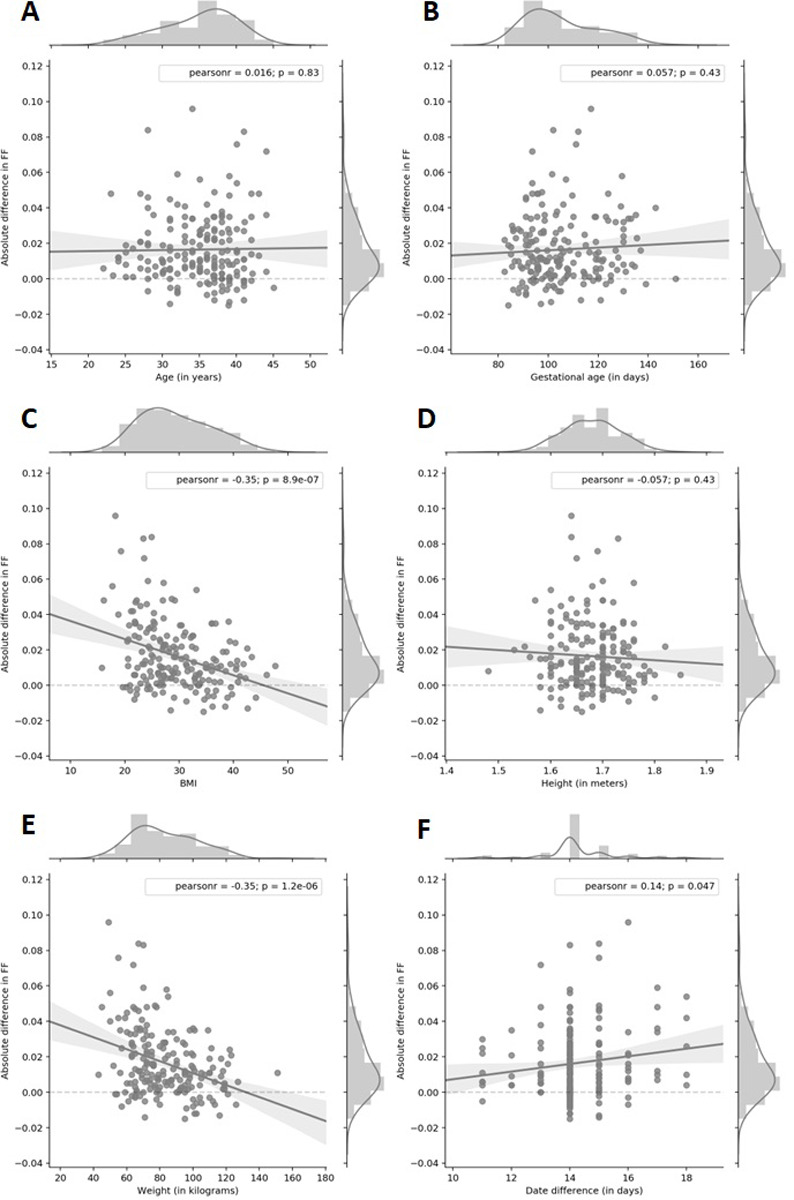
The association between change in fetal fraction and maternal age (A), gestational age (B), maternal BMI (C), height (D), weight (E) and date difference between blood draws (F) in repeated NIPT results due to uninformative first result.

In this part of the study, we examined a group of 189 pregnant women who initially received uninformative results due to low FF from cfDNA testing after first sampling. These women subsequently underwent a second blood sampling and analysis to obtain more conclusive results. After the second blood draw, the results in 112 cases (59.26%) were informative (all 112 cases were negative). In 77 cases (40.74%) the result was again uninformative. The median gestational age at the time of the initial blood draw was 14 weeks. In addition, the same result was found for the set of samples which were informative after the repeat analysis and also for those which were uninformative in both blood draws. The average time between the first draw and the second draw was 14.26, 14.35, and 14.14 days for all samples, informative after the second analysis, and uninformative after the second analysis, respectively (p-value = 0.55 from Kruskal-Wallis test). To further explore the factors that affect the success of repeated sampling, we considered the effects of maternal age, height, weight, BMI, gestational age, and time between blood draws (Fig 2). Our analysis demonstrated that the absolute change in FF between blood draws is significantly associated with maternal weight (Fig 2E—Pearson’s correlation coefficient = -0.35, p = 1.2e-06) and BMI (Fig 2C—Pearson’s correlation coefficient = -0.35, p = 8.9e-07) and not significantly associated with maternal age (Fig 2A—Pearson’s correlation coefficient = 0.016, p = 0.83), gestational age of the first sampling (Fig 2B—Pearson’s correlation coefficient = 0.057, p = 0.43), maternal height (Fig 2D—Pearson’s correlation coefficient = -0.057, p = 0.43), and time difference between samplings (Fig 2F—Pearson’s correlation coefficient = 0.14, p = 0.047).

#### Effect of gestational age on the fetal fraction

**Fig 3:**
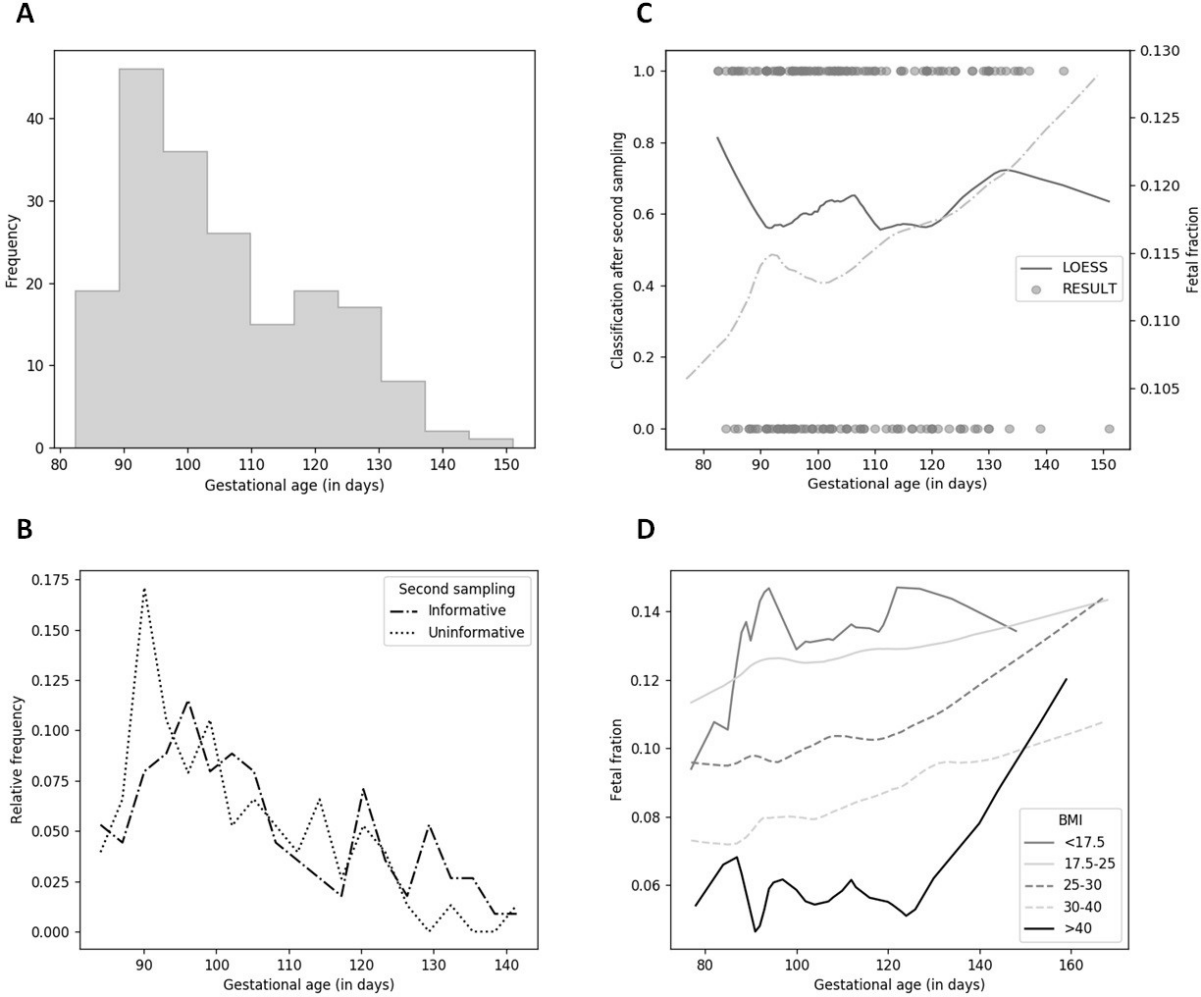
Effect of gestational age on the fetal fraction. (A) Histogram of gestational age for all 189 uninformative samples. (B) Distribution of informative/uninformative samples after the second sampling. (C) Loess-smoothed line for uninformative samples classified after second sampling to be informative (1)/uninformative (0). (D) Relationship between gestational age, BMI and fetal fraction. A model of fetal fraction versus gestational age and BMI was fitted, and the resulting lines are shown for various different BMI values.

There is a significant difference in the distribution for gestational age of uninformative samples that were informative after the second sampling (Fig 3B, dash-dotted line) and those that were uninformative after both sampling (Fig 3B). In the case of uninformative samples after the second sampling, a significantly higher representation can be observed at the sampling in the 13th gestational week (maximum reached at gestational day 89) compared to informative samples after the second sampling, which had the highest frequency one gestational week later (maximum reached at gestational day 96, Fig 3B). The relatively high frequency lasted up to the 16th gestational week (until gestational day 105), when there was a significant decrease in frequency. At the beginning of the 12th gestational week, it showed a higher frequency for the informative samples, which decreased at the end of this week.

The Loess data fitting method was used to observe the relationship between the classification of the samples after the second sampling (Loess smoothed line), FF (dash-dotted line), and the gestational age in the first sampling (Fig 3C). Zero represents samples classified after the second sampling as uninformative; informative samples after the second sampling are marked 1. The gestational age and level of FF refer to Fig 3C for the first sampling.

To further explore the variables that affect an informative redraw in different gestational days, we next considered the effect of maternal weight on FF (Fig 3D). We classified the women into five groups by maternal BMI based on the WHO obesity classification system. Generally, the importance of maternal weight was especially evident at the lowest and highest BMI values. Our results showed that median FF reached a maximum value at 92 days gestation and a minimum value at 102 days gestation in women with BMI < 17,5. We observed a positive increase in FF in three BMI groups (BMI = 17.5–25; 25–30; 30–40) on different gestational days. We also demonstrated a slightly decreasing trend in FF in the group with BMI > 40.

#### Prediction of informativeness

**Fig 4:**
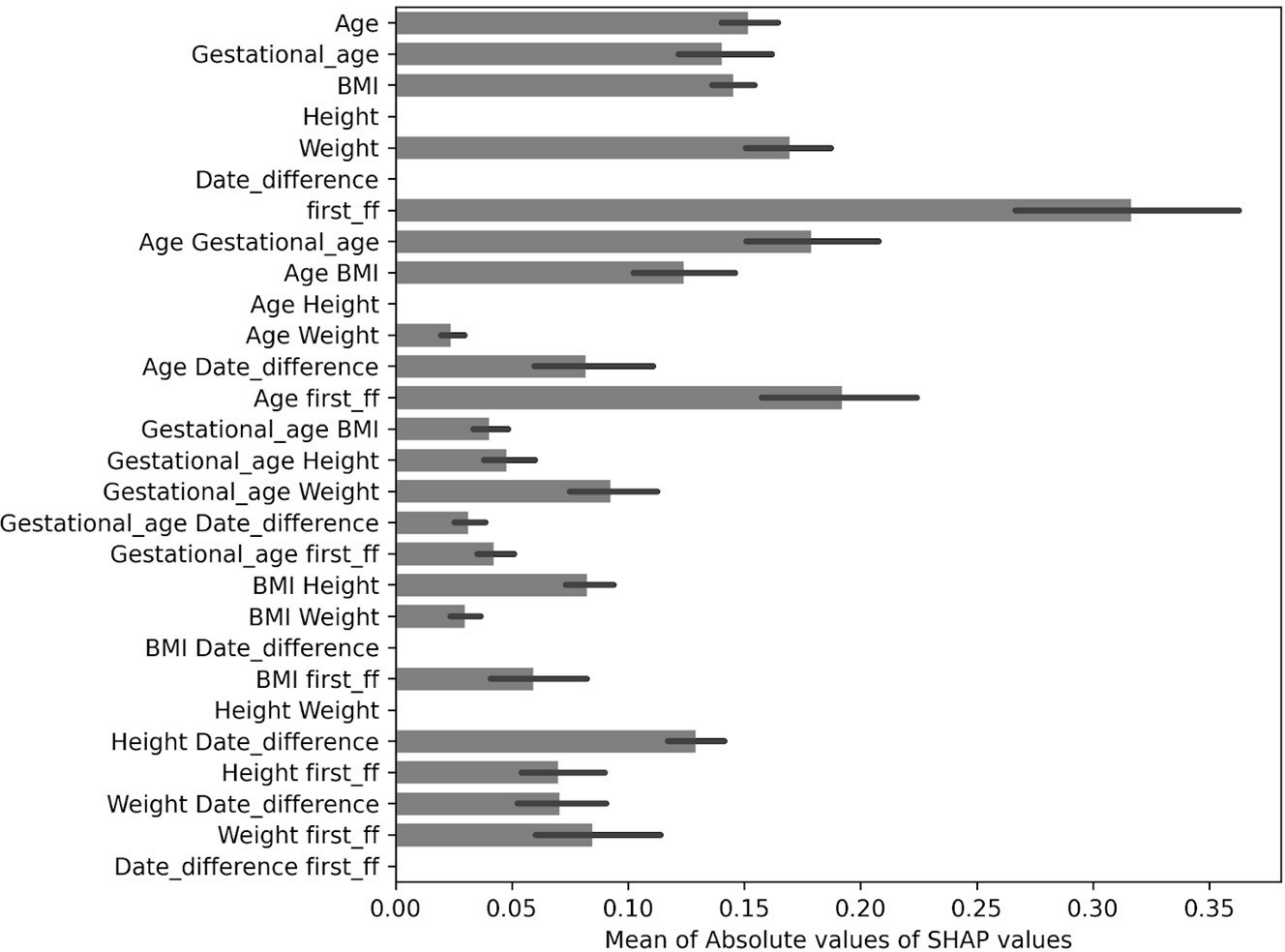
Summary of feature contributions calculated as mean of absolute SHAP values for test dataset. Length of each bar roughly represents the average importance of each attribute, with the bars corresponding to standard deviation of absolute SHAP values.

We tried to train a model for predicting the success of the second sampling based on known factors from the first sampling, i.e. Age, Gestational Age, BMI, Height, Weight, Time difference, and first_ff (FF after the first sampling). In Table 3 we show the performance of six models on training (132 samples) and validation (28 samples) sets. Despite the promising results on train and validation data, our final model choice (XGBoost) performed poorly on test data (29 samples), predicting samples with 58.6% accuracy (55.17% baseline test accuracy), 68.7% sensitivity, 49.15% specificity and 0.1447 Matthews correlation coefficient (MCC). The results did not improve even with the leave-one-out cross validation (loccv) testing method, whereby we concatenated the training, validation and testing datasets to increase the size of the training dataset. We trained a simple XGBoost model with default parameters for each partition. The model performed with 56.61% accuracy, 69.64% sensitivity, 37.66% specificity and 7.61% MCC. Adding interaction terms helped, where the XGBoost model (with the same set of parameters as the final model chosen from gridsearch) performed with 62.07% accuracy, 75% sensitivity, 46.15% specificity and 22.13% MCC. We calculated SHAP values for the model trained on a dataset with interaction terms to see how the attributes contributed to the final predictions with summarized results in Fig 4. SHAP values estimate the contribution of each attribute towards a final prediction of a datapoint. For example, a SHAP value of 0.2 for attribute “first_ff” (FF after the first sampling), means that the value of first FF for the datapoint contributed positively 0.2 towards final prediction.SHAP values can also be negative, and the final prediction can be inferred by taking the sum of all SHAP values from all the attributes for a datapoint. Summarizing this, by calculating mean of absolute values, helps discovering important predictors. The longer the bar, the higher on average was the contribution of the attribute on average (negative or positive), with error bars representing standard deviation. Most of the attribution agrees well with our other results, represented in the (S3 Fig).

**Table 3:** Metrics comparing tuned models for prediction of second sampling informativeness.

Model	Train Accuracy	Train Sensitivity	Train Specificity	Train MCC	Validation Accuracy	Validation Sensitivity	Validation specificity	Validation MCC
**Logistic Regression**	0,72	0,73	0,70	0,42	0,64	0,50	0,79	0,30
**LDA**	0,74	0,91	0,46	0,43	0,57	0,71	0,43	0,15
**QDA**	0,77	0,89	0,56	0,49	0,64	0,79	0,50	0,30
**SVM**	0,70	0,94	0,32	0,34	0,64	0,93	0,36	0,35
**Random Forest**	0,94	0,91	0,98	0,88	0,64	0,71	0,57	0,29
**XGBoost**	0,78	0,77	0,80	0,55	0,75	0,71	0,79	0,50

All models were slightly better than a baseline model predicting all samples as success in terms of accuracy (62.12% on train and 50% on validation). Matthews Correlation Coefficient (MCC) shows this as well, with values higher than zero, although still quite low, hinting that the chosen set of attributes is not sufficient with the amount of supplied data to provide confident and reliable insights.

Nevertheless, we still believe that with a larger dataset there is a space for improvement. The regression analysis showed similar results, where a constant baseline model was as good as a fitted Ridge regression model, with train RMSE of 0.017, validation RMSE = 0.018 and test RMSE of 0.021 (baseline RMSE = 0.021 for train and validation and 0.022 for test data). The R2 metric also shows that almost no variation was explained by the added features. Adding polynomial features did not help.

## Discussion

Although the NIPT is a screening test with a high specificity and sensitivity, a small percentage of tests fail due to low FF. In our study, we focused on a cohort of 5543 pregnant women with singleton pregnancy undergoing NIPT in Slovakia and identified 189 failed analyses after first sampling. Consistent with previous studies, our results showed that maternal weight and/or BMI are among the most significant factors influencing FF and, thus, the repeated NIPT failure. Consequently, we suggest the need for a specialized re-sampling management strategy for pregnant women based on BMI WHO categories. The low FF in pregnant women with BMI > 40 kg/m2 may be challenging to overcome by repeated sampling in the first trimester corresponding gestational age, and thus should be thoroughly considered. In addition, we assume that for women with a BMI under 25, it would be sufficient to prolong the time between sampling to overcome the problem of uninformative results due to low FF. The other two categories (BMI > 25 and < 40) do not need additional improvement due to the reasonably increasing increments of FF.

Of all the factors impacting the FF, maternal weight and/or BMI are the most significant predictor of test failure caused by an insufficient FF. A correlation between maternal weight and serum fetal DNA level was first demonstrated by Wataganara et al. in 2004 for pregnant women in the second trimester [30]. Wang et al. also confirmed a relationship between decreasing fetal cfDNA and increasing maternal weight (p = 0.0003), pointing out that 27% of the variations seen in the FF were explained by maternal weight and gestational age [10]. In addition, higher maternal weight has been reported to be associated with a low success rate of a repeated NIPT [10,31].

Our findings showed concordance with the previous studies. Multivariate analysis demonstrated that one additional kilogram translates to about a 6.5% increase in the risk of uninformative analysis. Our results also showed that maternal weight and/or BMI are very important factors that were significantly affecting repeated cfDNA test failure. Results from Pearson correlation analysis of FF and maternal BMI in all 5543 pregnancies showed a negative relationship. We also observed a gradually decreasing trend of FF in different BMI groups and, at the same time, a gradual increase in uninformative samples with increasing BMI (Fig 1). In accordance with our results and previous studies, BMI is one of the greatest factors which influences FF and therefore, NIPT success rate. Therefore, pregnant women with a higher BMI should be informed of the possible risk of a test failure. Compared with standard NIPT methods, sequencing shorter cfDNA fragments has recently been shown to be a reliable and promising method to reduce the probability of no calls results in obese pregnant women [32,33].

In our study, the range of maternal age was between 15 and 52 years, with a median of 35 years old. The present data from this population shows that maternal age is not a significant factor affecting repeated cfDNA test failure. However, results from Pearson correlation analysis of all 5543 pregnancies indicated a slightly significant decrease in FF with increasing maternal age. However, this phenomenon is due to the tendency of older women to gain weight, and so increase BMI (S4 Fig). When we grouped all samples into five groups of different ages, the FF gradually decreased with increasing maternal age and the relative frequency distribution for uninformative samples increased (Fig 1). Consistent with our results, Hou et al also reported that the percentage of FF significantly decreased with increasing maternal age [14]. Multivariate logistic regression analysis from 23 495 singletons and 928 twin pregnancies at 10 + 0 to 14 + 1 weeks’ gestation demonstrated that the risk of test failure increases by about 1.05 times with each additional kilogram of maternal weight and by about 1.02 times with each additional year of maternal age [15]. However, some studies found no significant association between FF and maternal age [8,34–37]. Thus, the effect of maternal age on the performance of NIPT has not reached an agreement and still needs further studies.

An additional factor that can also affect the FF is gestational age. In fact, many studies have confirmed that the FF is enhanced with the increase of gestational age. Wang et al. noted that levels of fetal cfDNA increased 0.1% per week between 10 and 21 weeks of gestation and after 21 weeks of gestation, fetal cfDNA increased 1% per week [10]. Benn et al. demonstrated that the probability that a repeated sampling will provide a result is dependent on the FF at the first draw, maternal weight, and time interval between draws, but is not strongly dependent on the gestational age at the time of the first sample [31]. Our results showed that there is a significant difference in the distribution of uninformative samples that were informative after the second sampling and those that were uninformative after both sampling (Fig 3B). Following this, we observed an apparently inverse relationship between the Loess-smoothed FF and the failure rate after the second sampling (Fig 3C). Using distribution FF on different days, we observed a different increase, possibly a decrease in FF in groups of women with different BMI (Fig 3D). We found that a repeating sample collection in women with a BMI >40 kg/m2 in the first trimester has only a minimal effect on overcoming the low FF and leads to a similar rate of test failure.

This study provides valuable clinical insights into the application of NIPS and diagnostic testing. Nevertheless, there are certain limitations that need to be addressed, and future research should concentrate on addressing these issues. One of the limitations is the absence of an evaluation of the relationship between FF and other factors, including serum-free β-hCG and PAPP-A. Another limitation pertains to the lack of data analysis regarding FF and positive test results. Additionally, it is important to highlight that our study lacks clinical data related to other maternal conditions, such as blood glucose and lipid levels, maternal autoimmune disease, low molecular weight heparin, as well as potential confounding variables, such as ethnicity, smoking, assisted reproductive technology conception, parity, excessive alcohol consumption, and dietary habits. Given the constraints within the current clinical dataset, there remains a significant need for further complex investigation into additional clinical factors that may influence FF.

The cutoff requirement for FF is dependent on the test provider. However, most testing laboratories require that the minimum FF is 4% [4]. Future improvements in technology may make it possible to obtain results at lower FFs. In the meantime, the findings of this study could form the basis for counseling parents concerning the likelihood of failure to obtain a result from NIPT. Further research is needed to define the biological variation in FF and identify factors that could potentially increase it.

## Supporting information

S1 Fig Graphical abstract of NIPT samples. (TIF)

S2 Fig Histogram of fetal fraction for all 5543 samples with male fetus. (TIF)

S3 Fig Comparison of FF of informative/non-informative samples and different attributes affecting FF. (A) Fetal fraction after first sampling (B) Date difference (in days) (C) Height (in meters) (D) Weight (in kilograms) (E) BMI (F) Gestational age (in days) (G) Age (in years).
(TIF)

S4 Fig Effect of maternal age on BMI. Figure shows a significant increase in BMI with increasing maternal age (Pearson’s correlation coefficient = 0.088, p = 5.6e - 11).
(TIF)

S1 Table Factors affecting cfDNA test failure (univariate and multivariate analysis). (DOCX)

S2 Table The median FF and number of informative/uninformative samples for each BMI and age categories. (DOCX)

S1 Dataset The anonymized data set necessary to replicate our study findings. (XLSX)
