# Supplementary figures and images for: Insights into non-informative results from non-invasive prenatal screening through gestational age, maternal BMI, and age analyses
Source: PLoS One. 2024 Mar 7;19(3):e0280858. doi: 10.1371/journal.pone.0280858 (PMC10919614; doi:10.1371/journal.pone.0280858)

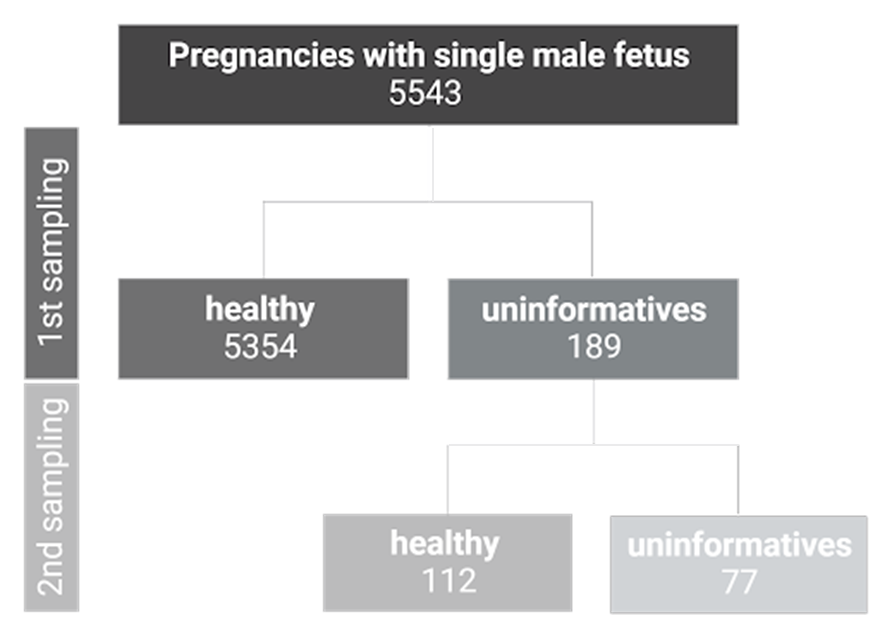

Supplement: S1 Fig — (TIF) [file pone.0280858.s001.tif]

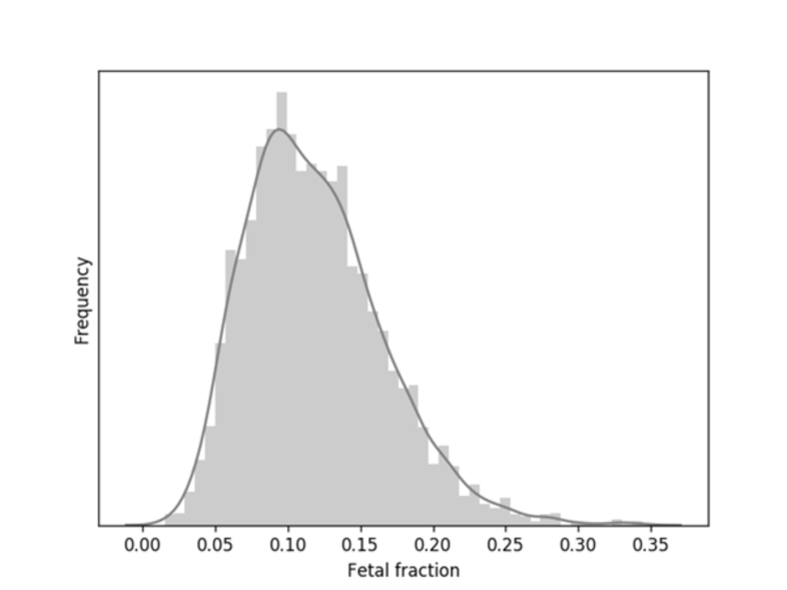

Supplement: S2 Fig — (TIF) [file pone.0280858.s002.tif]

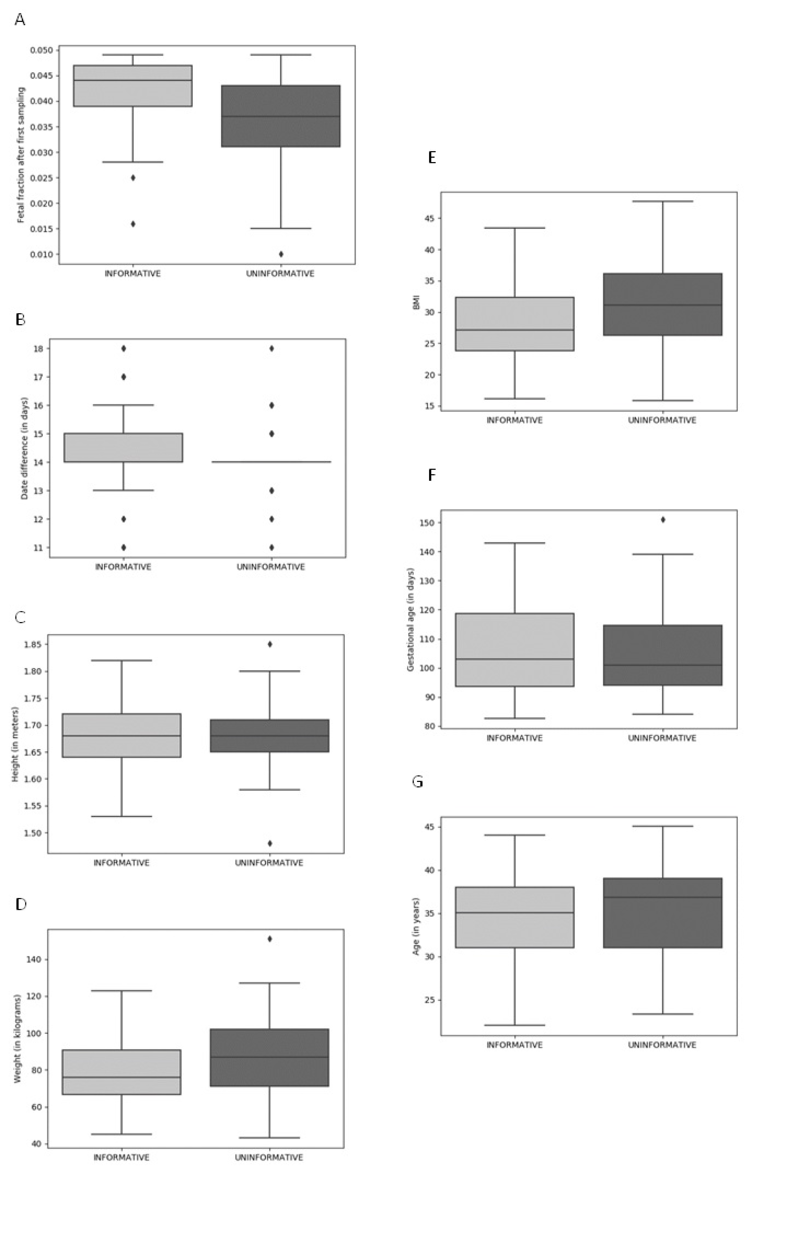

Supplement: S3 Fig — (A) Fetal fraction after first sampling (B) Date difference (in days) (C) Height (in meters) (D) Weight (in kilograms) (E) BMI (F) Gestational age (in days) (G) Age (in years). (TIF) [file pone.0280858.s003.tif]

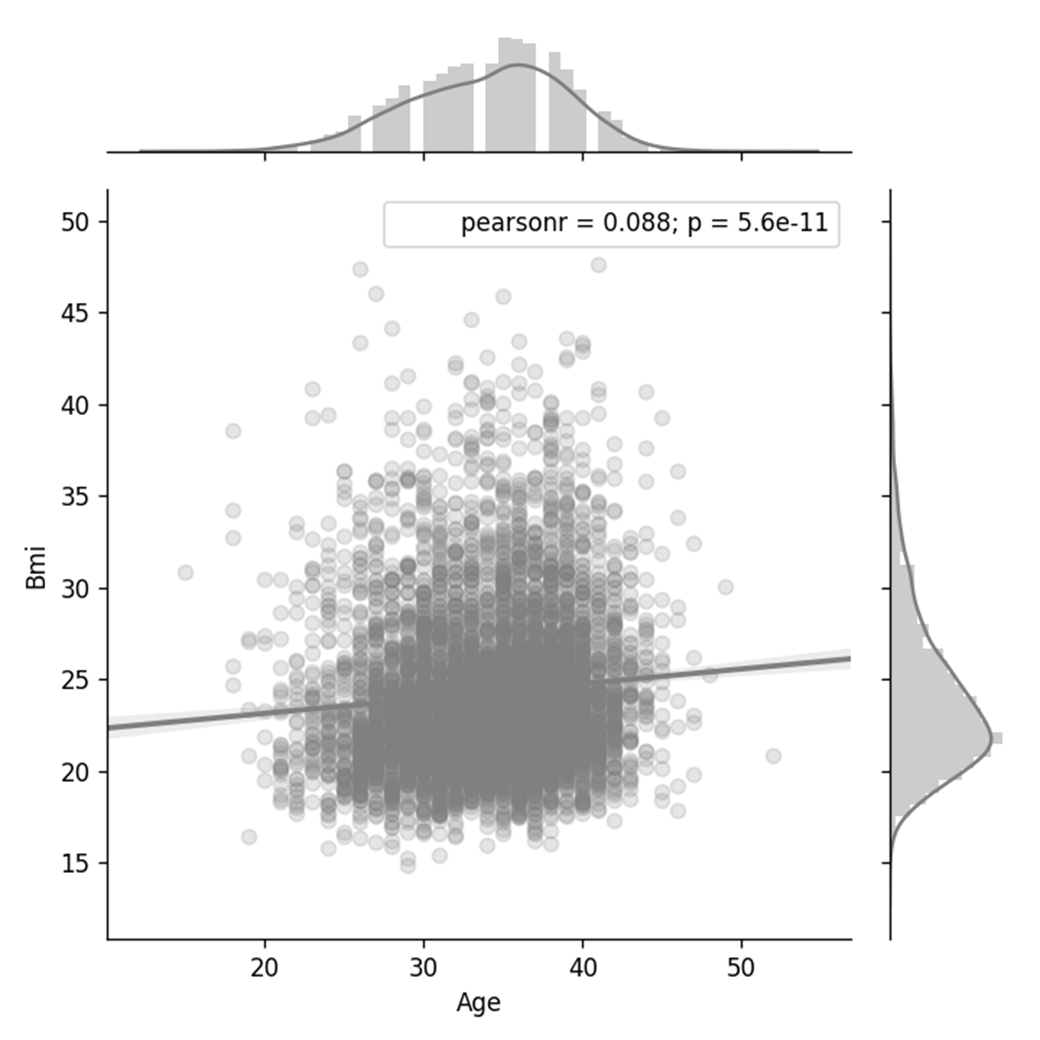

Supplement: S4 Fig — Figure shows a significant increase in BMI with increasing maternal age (Pearson’s correlation coefficient = 0.088, p = 5.6e - 11). (TIF) [file pone.0280858.s004.tif]
