# Supplementary material for: Insights into non-informative results from non-invasive prenatal screening through gestational age, maternal BMI, and age analyses
Source: PLoS One. 2024 Mar 7;19(3):e0280858. doi: 10.1371/journal.pone.0280858 (PMC10919614; doi:10.1371/journal.pone.0280858)
Supplement: S1 Table — (DOCX) [file pone.0280858.s005.docx]

In our study, 189 out of 5543 tests were uninformative due to low fetal fraction (3.4%). However, to be comparable with a previous study [15], we used only samples with gestational age lower than 14th week. Thus, we obtained 109 uninformative out of 2575 total tests (4.2%). We performed univariate logistic analysis of factors that affect the initial uninformative analysis. The logistic analysis showed good concordance with the previous study [15] except for gestational age and age of the mother which was statistically unimportant in our study probably due to low number of samples and/or limited range for gestational age. The odds ratios from the univariate analysis show that maternal weight is a very important factor and one additional kilogram translates to about a 7.0% increase (or 4.9% in [15]) in risk of uniformative analysis.

**Supplementary table 1A. Factors affecting cfDNA test failure (univariate analysis).**

| **Variable** | **Odds Ratio** [**^17^**](https://paperpile.com/c/5rTVb0/JJPRL) | **P** [**^17^**](https://paperpile.com/c/5rTVb0/JJPRL) | **Odds Ratio (our dataset)** | **P (our dataset)** |
| --- | --- | --- | --- | --- |
| **Maternal age in years** | 1.048 (1.033–1.064) | <0.0001 | 1.030 (0.989-1.072) | 0.153 |
| **Maternal weight in kg** | 1.041 (1.037–1.044) | <0.0001 | 1.070 (1.057-1.083) | <0.001 |
| **Maternal height in cm** | 1.001 (0.991–1.011) | 0.841 | 1.000 (0.968-1.033) | 0.990 |
| **Gestational age in weeks** | 0.872 (0.824–0.923) | <0.0001 | 0.856 (0.670-1.095) | 0.215 |
| **BMI kg/m^2^** | --- | --- | 1.219 (1.179-1.262) | <0.001 |
