# Supplementary material for: Insights into non-informative results from non-invasive prenatal screening through gestational age, maternal BMI, and age analyses
Source: PLoS One. 2024 Mar 7;19(3):e0280858. doi: 10.1371/journal.pone.0280858 (PMC10919614; doi:10.1371/journal.pone.0280858)
Supplement: S2 Table — (DOCX) [file pone.0280858.s006.docx]

**Supplementary table 2. The median FF and number of informative/uninformative samples for each BMI and age categories.**

Supplementary table 2A. The median FF and number of informative/uninformative samples for each BMI categories.

| **Maternal BMI range (kg/m2)** | **Median fetal fraction after first sampling (%)** | **Number of samples after first sampling** | **Number of uninformative samples after first sampling** |
| --- | --- | --- | --- |
| <17.5 | 13.1 | 51 (0.92%)* | 2 (3.9%)** |
| 17.5 - 25 | 12.4 | 3603 (65.0%)* | 54 (1.5%)** |
| 25 - 30 | 10.1 | 1250 (22.6%)* | 54 (4.3%)** |
| 30 - 40 | 8.2 | 599 (10.8%)* | 65 (10.9%)** |
| >40 | 6.1 | 40 (0.7%)* | 14 (35.0%)** |

*The percentages are calculated from the total number of pregnant women studied (5543).

**The percentages are calculated from number of samples after first sampling for each BMI categories.

Supplementary table 2B. The median FF and number of informative/uninformative samples for each age categories.

| **Maternal age range (years)** | **Median fetal fraction after first sampling (%)** | **Number of samples after first sampling** | **Number of uninformative samples after first sampling** |
| --- | --- | --- | --- |
| <25 | 11.6 | 267 (4.8%)* | 10 (3.7%)** |
| 25 - 30 | 11.6 | 1110 (20.0%)* | 27 (2.4%)** |
| 30 - 35 | 11.5 | 1848 (33.3%)* | 53 (2.9%)** |
| 35 - 40 | 11.0 | 1893 (34.2%)* | 77 (4.1%)** |
| >40 | 10.6 | 425 (7.7%)* | 22 (5.2%)** |

*The percentages are calculated from the total number of pregnant women studied (5543).

**The percentages are calculated from number of samples after first sampling for each age categories.
